# Thin-film ‘Thermal Well’ Emitters and Absorbers for High-Efficiency Thermophotovoltaics

**DOI:** 10.1038/srep10661

**Published:** 2015-06-01

**Authors:** Jonathan K. Tong, Wei-Chun Hsu, Yi Huang, Svetlana V. Boriskina, Gang Chen

**Affiliations:** 1 Department of Mechanical Engineering, Massachusetts Institute of Technology, Cambridge, MA 02139

## Abstract

A new approach is introduced to significantly improve the performance of thermophotovoltaic (TPV) systems using low-dimensional thermal emitters and photovoltaic (PV) cells. By reducing the thickness of both the emitter and the PV cell, strong spectral selectivity in thermal emission and absorption can be achieved by confining photons in trapped waveguide modes inside the thin-films that act as thermal analogs to quantum wells. Simultaneously, photo-excited carriers travel shorter distances across the thin-films reducing bulk recombination losses resulting in a lower saturation current in the PV cell. We predict a TPV efficiency enhancement with near-field coupling between the thermal emitter and the PV cell up to 38.7% using a thin-film germanium (Ge) emitter at 1000 K and an ultra-thin gallium antimonide (GaSb) cell supported by perfect back reflectors separated by 100 nm. Even in the far-field limit, the efficiency is predicted to reach 31.5%, which is over an order of magnitude higher than the Shockley Queisser limit of 1.6% for a bulk GaSb cell and a blackbody emitter at 1000 K. The proposed design approach does not require nanoscale patterning of the emitter and PV cell surfaces, but instead offers a simple low-cost solution to improve the performance of thermophotovoltaic systems.

A thermophotovoltaic system is a unique type of heat engine that directly converts thermal energy to electricity^1^,^2^,^3^,^4^. Typical TPV systems consist of a thermal emitter and a photovoltaic cell in which there are no moving parts allowing for a compact power generation platform. In theory, TPV systems can convert radiative energy from the thermal emitter to electricity at an efficiency approaching the Carnot limit for monochromatic radiation and can be used for both waste heat and solar energy harvesting^5^,^6^,^7^,^8^,^9^,^10^,^11^,^12^,^13^. However, the efficiency achieved in practical TPV systems has been limited by the mismatch between the thermal emission spectra and the PV cell absorption, thermalization of high energy charge carriers, and non-radiative recombination losses which are typically high in narrow band gap PV cells used for TPV.

To improve the efficiency, past studies have generally followed two approaches: (1) using intermediate frequency filters, such as rugate filters, photonic crystal filters, and plasma reflectors, which recycle low energy photons by returning them back to the thermal emitter and (2) engineering the emitter using rare-earth dopants, plasmonic metamaterials, and photonic crystals to achieve spectrally selective emission and angular selectivity^14^,^15^,^16^,^17^,^18^,^19^,^20^,^21^,^22^,^23^,^24^. By utilizing these approaches and minimizing system losses, several recent studies have demonstrated significant improvement to overall system efficiency for combustion and solar-based TPV systems^25^,^26^,^27^,^28^. In addition, previous studies have also exhibited TPV efficiencies, defined as the electrical power output divided by the net radiative heat input from the emitter to the PV cell, of nearly 24% by utilizing ternary, quaternary, and multi-quantum well cells at emitter temperatures of 1300–1500 K^29^,^30^,^31^,^32^. In parallel, other theoretical studies proposed utilizing the strong near-field coupling of electromagnetic fields between the emitter and PV cell separated by nanometer gaps to improve the efficiency and power density of TPV systems^33^,^34^,^35^,^36^,^37^,^38^,^39^,^40^. However, the proposed designs for high-efficiency near-field TPV systems required extremely narrow gap separations between the emitter and the PV cell, ultra-low band gap PV cells, and high emitter temperatures limiting practical realization.

In this work, we present a conceptually new approach to improve the spectral selectivity of a TPV system that is based on reducing the dimensions of the emitter and the PV cell in order to spectrally shape emission and absorption in analogy to the use of electronic quantum confinement effects in quantum wells^41^,^42^. In lieu of the electronic comparison, we define this approach henceforth as the ‘thermal well’ effect. The proposed design consists of a thin-film thermal emitter and a thin-film PV cell, which support quantized waveguide modes trapped inside the material by total internal reflection. The dispersion characteristics of these waveguide modes lead to resonant enhancement in the density of optical states (DOS) resulting in greater thermal emission and absorption especially in the near field^43^,^44^. More importantly, these waveguide modes also exhibit cut-off frequencies beyond which thermal radiation is suppressed since no mode is available for emission and absorption^45^,^46^. For an optimally thick thin-film emitter and PV cell, emission and absorption will be resonantly enhanced for photons with energies larger than the band gap of the cell and suppressed for photon energies smaller than the band gap. In this manner, the use of simple morphological structuring effects can dramatically improve the spectral selectivity of a TPV system. In addition, bulk non-radiative recombination losses are also reduced in the ultra-thin PV cell resulting in high internal quantum efficiencies (IQE) and a lower saturation current thus improving the electrical performance of the cell. The combination of these effects is predicted to result in a high TPV energy conversion efficiency at relatively low emitter temperatures. This effect can also be observed in both the far-field and near-field regimes where evanescent wave coupling of the trapped optical modes can further increase the conversion efficiency.

## Results

### Shaping Radiative Heat Transfer with Thermal Wells

**Figure 1:**
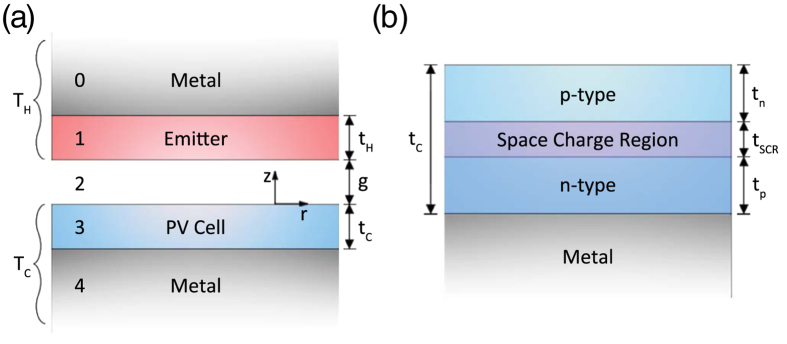
Schematics of the thin-film system studied for the radiative heat transfer model and the PV cell electrical model. (**a**) The TPV system consists of a thin-film emitter and a thin-film PV cell. Both films are placed onto semi-infinite back reflectors. (**b**) A magnified view of the PV cell detailing the structure assumed for the electrical analysis. The PV cell consists of a p-type quasi-neutral region, a n-type quasi-neutral region, and a space charge region. In this study, the thicknesses of the p-type and n-type quasi-neutral regions are assumed equal.

The TPV system considered in this study consists of two parallel thin-films, representing the emitter and the PV cell, which are separated by a gap g as shown in Fig. 1a. The emitter and the PV cell are at uniform temperatures of T_H_ and T_C_, respectively. The view factor is assumed equal to 1. It is also assumed that the materials of both the emitter and the cell have isotropic and local dielectric permittivities. Both of the thin-films are placed onto semi-infinite metallic back reflectors. In this work, back reflectors made of an ideal perfect metal and a real metal will be considered. The use of a perfect metal eliminates emission and absorption in the substrate thus providing a way to estimate the performance of the proposed TPV design due to only the thermal well effect. These results can be directly compared to the previously studied case of a bulk emitter and a bulk PV cell to highlight the benefits of morphological structuring.

In this study, thermal radiation is modelled using a rigorous analytical electromagnetic formulation based on Rytov theory (see Methods)^47^,^48^,^49^. Several configurations are evaluated to assess the potential of using thin-film emitters and absorbers to improve TPV performance. For all cases, a GaSb PV cell with a band gap of 0.726 eV is used, which is conventional in many TPV platforms^50^,^51^,^52^,^53^. To demonstrate the thermal well concept, we chose a thin-film of Ge as the thermal emitter. Ge is a high refractive index semiconductor with a band gap of 0.7 eV. The material absorption in Ge at wavelengths above the band gap naturally provides strong emission channels by virtue of Kirchoff’s law. By combining the spectral properties of bulk Ge absorption with morphological structuring of the emitter, it is possible to spectrally tailor thermal emission for TPV systems.

We also considered a bulk W emitter in order to compare thermal emission spectra of waveguide modes confined in Ge thermal wells with previously studied emission of surface plasmon polariton (SPP) modes thermally excited on the W surface^35^. W is a refractory metal that is thermally stable at high temperatures and supports SPP modes in the near infrared wavelength range. SPP modes are resonant surface waves that form between materials with dielectric permittivities of opposite sign. These modes exhibit significant enhancement in DOS and hence radiative transfer when in close proximity to the surface^35^,^43^. However, due to the highly confined nature of SPP modes, this enhancement in DOS decreases rapidly away from the surface. Nevertheless, W emitters are still used in more conventional TPV systems because of its low plasma frequency^54^.

The backside metal supporting the emitter and the PV cell is chosen to be either a perfect metal (PM), silver (Ag) on the PV cell side, or tungsten (W) on the emitter side. When both W and Ag are used, a magnesium fluoride (MgF_2_) spacer is placed between the GaSb cell and the Ag mirror in order to minimize coupling of SPP modes in W and Ag in the near-field regime. The optical constants of GaSb, Ge, MgF_2_, Ag and W were obtained from literature^55^. The infrared optical properties of Ag were extrapolated using a Drude model.

**Figure 2:**
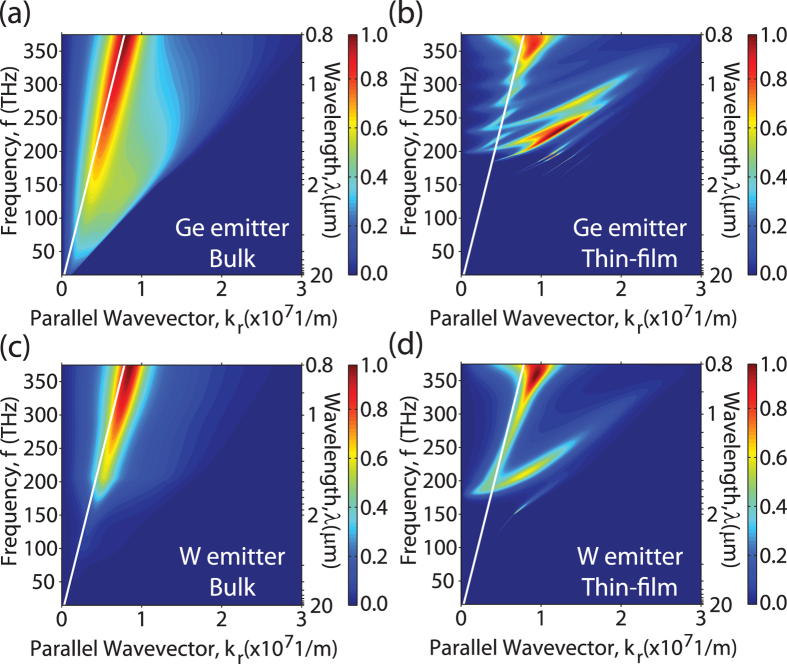
The normalized transmission function, G_NT_, comparing a bulk system to a thin-film system for different emitter materials and a GaSb cell: (**a**) a bulk Ge emitter and bulk GaSb cell, (**b**) a thin-film Ge emitter and a thin-film GaSb cell. The thin-film thicknesses are t_H_ = 860 nm and t_C_ = 136 nm, (**c**) a bulk W emitter and a bulk GaSb cell, (**d**) a bulk W emitter and a thin-film GaSb cell. The thin-film thickness is t_C_ = 134 nm. This comparison clearly showcases the effect of morphology on the trapped optical modes available for radiative transfer. The gap distance is assumed to be g = 100 nm. The light line is also plotted to differentiate propagating modes (above the light line) and evanescent modes (below the light line).

To demonstrate the effect of reducing a bulk system to a thin-film structure, Fig. 2a,b shows the normalized transmission function, G_NT_, for a bulk Ge emitter and a bulk GaSb cell and their corresponding thin-film counterparts, respectively. For the thin-film thermal well structure, a perfect metal was assumed to be on the backside of both the emitter and the PV cell. The optimum film thicknesses that maximize the TPV efficiency were obtained assuming an emitter temperature of 1000 K, a PV cell temperature of 300 K, and a gap separation of 100 nm. The emitter temperature was chosen to be below the melting temperature of Ge which is nearly 1200 K. As shown in Fig. 2, the transmission function for the bulk system indicates that radiative modes are supported over a broad frequency range. This corresponds to the n^3^ enhancement in the bulk photon DOS of high refractive index media^43^. In contrast, the transmission function for the thin-film structure changes dramatically and is now comprised of several distinct bands which indicate the presence of trapped waveguide modes in both the Ge emitter and GaSb cell.

Depending on the coupling strength between these waveguide modes, radiative transfer can either be enhanced or suppressed. Stronger coupling occurs when the waveguide modes supported in the emitter and the PV cell overlap in both frequency and in-plane wavevector and vice-versa for weaker coupling. Therefore, by choosing appropriate thicknesses for the thin-film emitter and PV cell, it is possible to simultaneously enhance thermal radiation at wavelengths above the GaSb band gap and suppress thermal radiation at wavelengths below the band gap as shown in Fig. 2b. Between wavelengths of 1.1 μm and 1.6 μm, a strong band exists which indicates similar waveguide modes are supported in both the emitter and PV cell resulting in strong coupling and thus large heat flux. However, at wavelengths shorter than 1.1 μm and wavelengths longer than 1.6 μm several weaker bands can be observed. This is due to the difference in thicknesses between the emitter and PV cell where the more numerous waveguide modes supported in the emitter can only weakly couple to the few modes that are above the cut-off frequencies supported by the PV cell in this wavelength range.

Although this mismatch in waveguide modes also reduces the relative coupling strength for above band-gap modes, the suppression of sub-band gap radiative transfer is more crucial in improving TPV performance for a Ge emitter and GaSb cell combination. Furthermore, since GaSb and Ge are both semiconductors with similar band gaps, both materials exhibit an increase in the imaginary component of the permittivity at wavelengths above the band gap. This results in the broadening of the waveguide modes which allows the increased radiative energy transfer from the emitter to the PV cell. Although the mechanism of tailoring spectral thermal emission from low-dimensional structures is general and applicable to any material combination of the emitter and the PV cell, the optimum thermal well thicknesses are specific to a particular combination of materials and operating temperatures.

For comparison with previously proposed near-field TPV designs, a bulk W emitter was also calculated. Figure 2c,d show the corresponding transmission functions for a bulk and a thin-film GaSb cell, respectively. Once again, the transmission function for the bulk system has a broad spectrum similar to Fig. 2a. In the thin-film case, we can still observe the formation of distinct bands. When compared to Fig. 2b, fewer bands are observed which indicates that these bands are solely due to the waveguide modes in the PV cell. Similar to Fig. 2b, a distinct band can once again be observed from 1.1 μm to 1.6 μm. The peak at 950 nm is due to the surface plasmon polariton modes supported in the W emitter.

**Figure 3:**
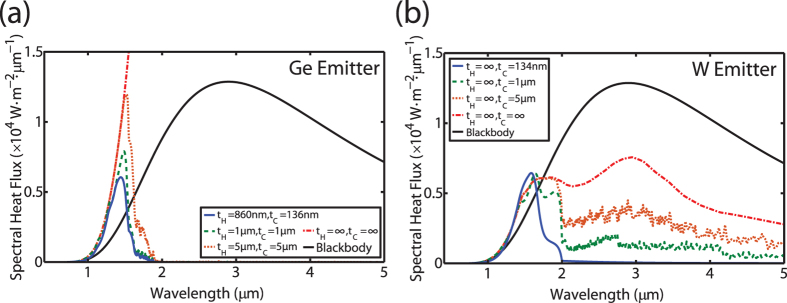
The spectral heat flux as a function of the emitter and the PV cell thicknesses assuming an emitter temperature T_H_ = 1000 K and a gap distance g = 100 nm: (**a**) the spectral heat flux for a Ge emitter, (**b**) the spectral heat flux for a W emitter. By making the emitter and the PV cell thin, radiative energy transfer at wavelengths below the band gap is significantly suppressed.

Figures 3a,b show the spectral heat flux for a Ge emitter and W emitter, respectively, again for the case that the back reflector is a perfect metal. For both cases, the emitter temperature was assumed to be 1000 K and the gap separation assumed to be 100 nm. In Fig. 3a, a progressive decrease in the thickness of the emitter and the PV cell results in strong spectral shaping of the heat flux where long wavelength thermal radiation is significantly suppressed and short wavelength thermal radiation is enhanced resulting in thermal radiation higher than the blackbody limit at the same temperature. This behavior is expected as photonic confinement effects only occur when the thickness of the thin-films are comparable to or smaller than the wavelength of IR radiation. In this regime, the cut-off frequencies of the waveguides modes will blue shift as the thickness decreases. Therefore, long wavelength radiation is inherently more sensitive to variations in thickness. By comparison, the short wavelength range is relatively insensitive to variations in thickness and from Fig. 3a it can be observed in that even for thicknesses of 5 μm, the spectral heat flux overlaps with the bulk structure at wavelengths shorter than 1.5 μm.

For the case of a bulk W emitter, Fig. 3b shows that as the thickness of the GaSb cell decreases, long wavelength thermal radiation is still suppressed while short wavelength thermal radiation is again enhanced beyond the blackbody limit. This suggests that despite the broad emission of SPP modes, the thermal well effect can still be utilized to dramatically improve the spectral selectivity by simply making the PV cell thin in order to suppress absorption at longer wavelengths.

At this point, it is worth mentioning that Ge at high temperatures will also exhibit a broader emission spectrum due to a combination of band gap shrinkage and free carrier emission. For the sake of demonstrating the thermal well concept, this effect was not included in the results plotted in Figs. 2 and 3. Although neglecting this effect may appear to be an oversimplification, the results for the bulk W emitter indicate that despite the presence of long wavelength emission, the inability of the GaSb cell to absorb thermal radiation in this wavelength range will ensure that the spectral selectivity of the TPV system is maintained. To confirm this assumption, additional calculations were performed in which the extinction coefficient of Ge was artificially raised to emulate the effects of high temperature and the results show that the spectral selectivity was still improved via the thermal well effect (see Supplementary Information for further details).

### Improving TPV System Performance using Thin-Film PV Cells

To assess the TPV conversion efficiency of the system we combine the radiative heat transfer model with an electrical model that considers the recombination of charge carriers in the PV cell (see Methods). This model is based on previous studies and treats the PV cell as a diode which consists of a p-type quasi-neutral region, n-type quasi-neutral region, and a space charge region as shown in Fig. 1b. 35,56,57,58 Bulk and surface recombination effects are included in order to provide a more realistic estimate of device performance compared to the Shockley Queisser limit. However, in this analysis, we assume that the internal quantum efficiency (IQE) is 100%. As will be shown, this assumption is appropriate when considering ultra-thin PV cells with negligibly thin p-type and n-type quasi-neutral regions.

In order to assess the impact of the emitter and cell morphology on TPV system performance, a comparison can still be made between the efficiencies for the thin-film structures with a maximum efficiency calculated from the Shockley Queisser formulation which assumes no non-radiative recombination processes in the PV cell and only far-field radiative transfer occurs^59^. If the emitter is a blackbody at a temperature of T_H_ = 1000 K and the PV cell is at temperature of T_C_ = 300 K, the Shockley Queisser formulation predicts the maximum efficiency achievable for a GaSb cell is 1.5%. For an emitter temperature of T_H_ = 2000 K, the efficiency increases to 20.7%.

**Figure 4:**
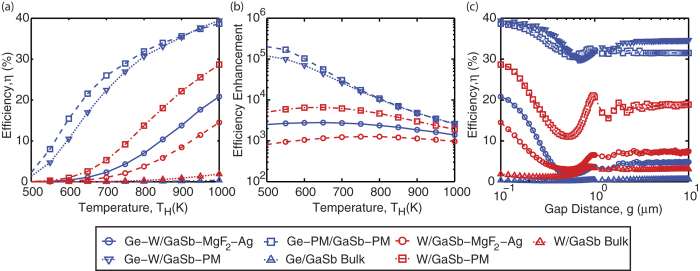
The energy conversion efficiency, η, from the emitter to the PV cell for several material combinations. (**a**) The efficiency as a function of temperature assuming a gap distance of g = 100 nm. (**b**) The predicted efficiencies from (**a**) normalized by efficiencies computed using the Shockley Queisser formulation for varying blackbody emitter temperatures. (**c**) The efficiency as a function of gap distance assuming an emitter temperature T_H_ = 1000 K. The legend is identical to (a). The optimal thicknesses for the case of a Ge emitter on a W substrate and a GaSb cell on a Ag substrate with a MgF_2_ spacer is t_H_ = 119 nm, t_C_ = 100 nm, and t_S_ = 1.25 μm. The optimal thicknesses for a Ge emitter on a W substrate and a GaSb cell on a perfect metal are t_H_ = 58 nm and t_C_ = 94 nm. The optimal thicknesses for a bulk W emitter and a GaSb cell on a Ag substrate with a MgF_2_ spacer is t_C_ = 59 nm and t_S_ = 750 nm.

Using the calculated radiative power spectra and equations (3, 4, 5, 6, 7, 8, 9) (see Methods), the predicted efficiencies for the thin-film structure, which again includes non-radiative bulk and surface recombination losses, are shown in Fig. 4a as a function of temperature assuming a gap separation of 100 nm. At an emitter temperature of T_H_ = 1000 K, the predicted efficiencies in the bulk limit is 0.38% for a Ge emitter and 1.9% for a W emitter due to the broad spectrum of radiative heat transfer shown in Fig. 3. By applying the thermal well effect and reducing the dimensionality of the emitter and PV cell, the energy conversion efficiency can be improved dramatically. For the case where perfect metals are used for both back reflectors, the efficiency reaches 38.7% for a thin-film Ge emitter and 28.7% for a bulk W emitter, which is more than an order of magnitude higher than the bulk limit. These predicted efficiencies also exceed past TPV efficiency records of 22% for GaSb cells while using a substantially lower emitter temperature of 1000 K compared to temperatures higher than 1500 K^9^,^60^.

To investigate this enhancement further, if the perfect metal back reflector on a thin-film Ge emitter is replaced with W, it can be observed that at low temperatures the efficiency is lower compared to the case of a perfect metal due to broadening of thermal emission at longer wavelengths. However, at higher temperatures, shorter wavelength modes are preferentially excited as the Planck energy oscillator function blue shifts. As a result, the short wavelength SPP mode supported in W contributes more to radiative transfer compensating for the long wavelength emission. In conjunction with thermal emission from the thin-film Ge emitter, this combination can actually lead to an even higher efficiency of 39.4% again at T_H_ = 1000 K.

For a more realistic TPV system, where the back reflector is replaced with a Ag substrate separated from the PV cell by a transparent MgF_2_ spacer layer, the efficiency decreases to 20.8% for a thin-film Ge emitter supported by a W substrate and 14.5% for a bulk W emitter at T_H_ = 1000 K. This reduction in performance is due to the use of W and Ag, which not only support SPP modes that can couple in the near-field regime, but also exhibit intrinsic parasitic absorption and emission at longer wavelengths due to the imperfect nature of these materials as back reflectors. Although the MgF_2_ spacer layer reduces SPP mode coupling by increasing the distance between the emitter and the PV cell back reflectors, long wavelength absorption and emission still inhibit the predicted performance. This suggests that bulk metallic mirrors are not suitable to fully harness the thermal well effect. Despite the reduction in performance, the predicted efficiency still exceeds the bulk limit. Thus, even for a suboptimal system, the thermal well effect can still provide dramatic improvements to TPV performance.

In fact, the predicted efficiencies for all cases not only exceed the bulk limit, but also the Shockley Queisser limit for a blackbody emitter by several orders of magnitude. Figure 4b shows the predicted efficiency normalized to the efficiency from the Shockley Queisser formulation assuming the emitter is a blackbody and the PV cell has a band gap of 0.726 eV. For certain cases, the enhancement reaches its maximum at intermediate temperatures. This is due to the more sensitive nature of thermal emission in the near-field regime compared to blackbody emission where lower temperatures will red shift the population of radiating modes resulting in a more rapid decrease in thermal emission at wavelengths where strong evanescent coupling occurs. The data presented in Figs. 3a,b demonstrate that the use of the thermal well effect can enable operation of near-field TPV systems with efficiencies higher than 30% at moderate temperatures of about 800 K.

Due to the inherent practical difficulties associated with utilizing the near-field regime in a TPV system, the conversion efficiency was also calculated as a function of the gap separation assuming an emitter temperature of T_H_ = 1000 K as shown in Fig. 4c. For all cases, the efficiency saturates at gap separations larger than 5 μm which corresponds to the far-field limit. In this limit, for the case where both back reflectors are perfect metals, the efficiency decreases to 31.5% for a thin-film Ge emitter and 18.9% for a bulk W emitter. For the case of a thin-film Ge emitter and a W back reflector, the efficiency decreases to 34.6%. This reduction in performance is expected as the enhancement by near-field coupling is no longer utilized. However, despite this reduction, the predicted efficiencies still clearly exceed the Shockley Queisser limit for a blackbody emitter. The oscillatory behavior of efficiency at intermediate gap separations is due to the vacuum gap behaving like a waveguide.

In the case where a Ag back reflector and a MgF_2_ spacer on the PV cell are used, the efficiency decreases more significantly to 4.9% for a thin-film Ge emitter supported by a W substrate and 7.4% for a bulk W emitter. Again this can be attributed to parasitic emission and absorption in W and Ag which lead to significant radiative transfer at longer wavelengths which cannot be used for power generation.

## Discussion

The enhancement in TPV system efficiency, as demonstrated in Fig. 4, can be attributed to improvements to both the spectral selectivity of radiative transport via the thermal well effect, as was shown in Fig. 3, and a reduction in bulk recombination losses for a thin-film PV cell which results in a lower saturation current. It should be emphasized that although the thermal well effect was proposed as a way to manipulate the photon DOS in the near-field regime, it can also be used to improve the spectral selectivity in the far-field limit as evidenced by the high efficiencies predicted for the cases that use a perfect metal as a back reflector in Fig. 4c. Again this can be attributed to the creation of quantized waveguide modes in the emitter and the PV cell. Although resonant enhancement for high photon energies larger than the band gap of the PV cell will be weaker due to the lack of near-field coupling, the suppression of emission and absorption below the cut-off frequency of these waveguide modes still ensures strong spectral selectivity in the far-field regime.

In regards to the electrical performance of the GaSb cell, the thickness of the space charge region formed is estimated to be t_SCR_ = 135 nm based on equation (9) (see Methods) and the properties of a typical GaSb cell. For the various cases presented, the PV thin-film thicknesses are either comparable to or smaller than the predicted size of the space charge region. This implies that the p-type and n-type quasi-neutral regions in the PV cell are so thin that bulk recombination losses are negligible. Surface recombination losses can also be dramatically reduced if the PV cell is well passivated. By significantly reducing these loss mechanisms, ultra-thin PV cells can exhibit an IQE that approaches 100% thus justifying our earlier assumption^56^,^58^. Furthermore, the saturation current will also decrease resulting in a higher open-circuit voltage. Both of these effects contribute to improve the energy conversion efficiency.

To show the impact of reducing bulk recombination losses on the PV performance, the predicted efficiency was also calculated using the Shockley-Queisser formulation assuming the same radiative power density for each case (see Supplementary Information). When both back reflectors are perfect metals, the predicted efficiencies at an emitter temperature of T_H_ = 1000 K and a gap separation of g = 100 nm are 46.4% and 34.5% for a thin-film Ge emitter and a bulk W emitter, respectively. For a thin-film Ge emitter supported by a W back reflector, the efficiency is 46.7%. And finally, for the PV cell back reflector composed of a Ag mirror and a MgF_2_ spacer, the efficiency is 25.2% and 17.4% for a thin-film Ge emitter and a bulk W emitter, respectively. In all cases, the efficiencies calculated using the more realistic electrical model approach the performance predicted by the ideal Shockley Queisser formulation. Therefore it is clear that by using thin-film PV cells, the corresponding reduction in bulk recombination losses can dramatically improve the electrical performance of the device.

It was also observed that by combining different emitting mechanisms, namely thin-film waveguide modes in Ge and SPP modes in W, it is possible at high temperatures to exceed the efficiency predicted using a perfect metal as the back reflector on the emitter. This suggests that there exists some flexibility in the design of the emitter and with an optimal material combination for the thin-film and substrate, even higher energy conversion efficiencies can be obtained compared to the predictions in this study. However, to achieve high energy conversion efficiencies the back reflector of the PV cell must exhibit a near unity reflectance to eliminate parasitic absorption and emission at photon energies smaller than the band gap of the PV cell. By replacing the perfect metal with a Ag substrate and a MgF_2_ spacer, the performance was observed to decrease significantly, which indicates that bulk metallic mirrors are insufficient in this design approach. In general, no natural materials behave like a perfect metal; however, it is possible to design artificial photonic structures that can emulate the behavior of a perfect metal within a certain wavelength range. For example, a distributed Bragg reflector, which is commonly used as a high quality mirror, supports a photonic band gap, which can be positioned at photon energies just below the electronic band gap of the PV cell^61^,^62^. In fact, the concept of incorporating a Bragg reflector in a PV module is not new and has been used in the past as a way to recycle photons in various PV cells^63^,^64^,^65^,^66^,^67^,^68^. Although it is impossible to completely eliminate metal, since electrical contacts are needed to extract charge carriers, a recent study has shown that it is ideal to minimize the contact area between the PV cell and the metal contacts to reduce recombination losses^69^. Therefore, the inclusion of a Bragg mirror could provide a path towards realistically achieving TPV performance that approaches the perfect reflector case presented in this work. To showcase the potential of using a dielectric mirror, a preliminary design was developed which exhibits a reflectance near unity at photon energies just below the band gap of the GaSb cell (see Supplementary Information for further details).

To improve the performance of the TPV system even further, it should be stressed that the choice of the materials in this study are not the most optimal. For example, the band gap of the GaSb cell is relatively large compared to the low emitter temperatures thus inhibiting the portion of thermal radiation that can be used for power generation. Smaller band gap ternary and quaternary PV cells, such as InGaAsSb or InGaSb devices, can extend the range of useful photons for power generation to longer wavelengths resulting in both a higher efficiency and a higher electrical power density at lower emitter temperatures. In fact, given that the optimal PV cell thickness in this study is significantly thinner than typical epitaxially grown ternary and quaternary devices, it is likely that TPV systems utilizing these materials will benefit from the thermal well effect^14^,^70^,^71^,^72^. Furthermore, the inherent simplicity of reducing the dimensionality of the emitter and the PV cell allows conventional components such as filters or antireflection coatings to be easily incorporated which can improve the spectral selectivity even further. For practical realization, additional studies are needed to evaluate the thermal well effect for a more realistic PV device architecture that includes passivation, electrical contacts, as well as 2D and 3D carrier transport. Thermal effects on TPV performance due to thermalization losses and parasitic heating should also be evaluated; though, it should be noted that by using the thermal well effect in conjunction with a PV cell with an optimal band gap, it may be possible reduce heating compared to conventional PV cells^57^.

By improving the spectral selectivity and reducing the saturation current, the studied thermal well effect is theoretically predicted to enhance the energy conversion efficiency of TPV systems by more than an order of magnitude compared to both the bulk limit and the Shockley Queisser limit for a blackbody emitter at the same temperature. For a thin-film Ge emitter and a thin-film GaSb PV cell supported by perfect metals, the TPV energy conversion efficiency was predicted to be as high as 38.7% at an emitter temperature of 1000 K and gap separation of 100 nm due to only the thermal well effect. In the far-field limit, this efficiency decreases to 31.5%; however, this is still significantly higher than the Shockley Queisser limit even for a blackbody emitter at a temperature of 2000 K. This is in stark contrast to past studies that utilized SPP modes to improve TPV systems as the efficiency enhancement is limited to the near-field regime which is challenging to realize in a practical system.

Overall, thermal well TPV systems can provide higher TPV efficiency at lower emitter temperatures much like quantum well lasers which feature lower pumping thresholds than conventional diode lasers while being exceedingly more efficient or quantum well thermoelectric generators, which have thermoelectric figures of merit that are much higher than bulk systems^73^. By introducing morphological effects into a TPV system, not only can greater flexibility in engineering radiative heat transfer between the emitter and the PV cell be achieved, but also even simple, easily fabricated structures can have a profound effect on the overall performance of the TPV system.

## Methods

### Rytov Theory

We model the energy exchange between the thermal emitter and the PV cell using a rigorous analytical electromagnetic formulation based on Rytov theory, in which thermally emitting bodies are modelled as a volume of fluctuating dipole sources whose amplitude is determined by the fluctuation dissipation theorem^47^,^48^,^49^,^74^,^75^,^76^,^77^,^78^. Dyadic Green’s functions are then used to calculate the Poynting vector at a position relative to the emitting body. In this manner, the rate of radiative energy transfer, or heat flux, can be determined from one medium to another. It should be noted that this formalism is valid in both the near-field and far-field regimes. The general form of the heat flux is as follows,


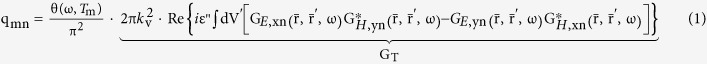


where q_mn_ is the heat flux from medium m to n, θ is the energy for a Planck oscillator, and G_E_/G_H_ are the dyadic Green’s functions for the electric and magnetic fields^79^. The term G_T_ is defined as the transmission function and is dependent on both frequency and the in-plane wavevector of photons participating in the radiative heat exchange. The transmission function represents the available radiative channels for energy transport between the emitter and PV cell. Therefore, any modification of the photon DOS imposed on the system by changing its geometry and/or material will manifest itself directly in the transmission function.

In order to utilize equation (1) to find the heat flux, specific solutions to the dyadic Green’s functions must be found for a particular geometry. For the system defined in Fig. 1, the analytical solutions can be found by using a combination of the transfer matrix and scattering matrix methods^80^. Further details of the formulation can be found in the supplementary information. With this formulation, the heat flux into a particular layer is determined as the difference between the incoming and outgoing Poynting vectors for that layer. Therefore, the net heat flux into the PV cell, q_ω_, can be determined as follows,


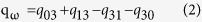


where q_03_ is the heat flux from the emitter back reflector to the PV cell, q_13_ is the heat flux from the thin-film emitter to the PV cell, q_31_ is the heat flux from the PV cell to the thin-film emitter, and q_30_ is the heat flux from the PV cell to the emitter back reflector. By using equations (1) and (2), the radiative power spectrum absorbed by the PV cell can be calculated for a particular structure and set of materials.

### TPV System Efficiency Calculation

In order to provide a more realistic estimation of the TPV system performance compared to the more idealized Shockley Queisser formulation, we incorporated specific material recombination lifetimes to account for both radiative and non-radiative recombination losses. Following the conventional definition for the photovoltaic energy conversion efficiency, we define the efficiency of our TPV system as^56^,^57^,^58^,^59^,^81^,^82^,


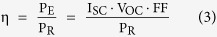


where η is the TPV energy conversion efficiency, P_E_ is the electrical power density, P_R_ is the radiative power density, FF is the fill factor, I_SC_ is the short-circuit current, and V_OC_ is the open-circuit voltage. In this model, the short-circuit current is assumed to be approximately equal to the photo-generated current, I_PH_. The radiative power density is the net radiative heat transfer between the emitter and the PV cell integrated over all frequencies,


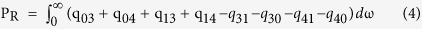


where q_04_ is the heat flux from emitter back reflector to the PV cell back reflector, q_14_ is the heat flux from the thin-film emitter to the PV cell back reflector, q_41_ is the heat flux from the PV cell back reflector to the thin-film emitter, and q_40_ is the heat flux from the PV cell back reflector to the emitter back reflector. To calculate the short-circuit current, I_SC_, the power spectrum is integrated for photon energies larger than the band gap of the PV cell. By assuming each absorbed photon produces one electron, the short-circuit current can be obtained as follows,


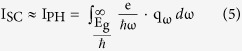


In equation (5), we assume the IQE is 100%. Normally this will lead to an overestimation of PV performance. However, in the limit of ultra-thin PV cells, this assumption is a reasonable approximation since bulk recombination losses for minority carriers are significantly reduced if the p-type and n-type regions are negligibly thin. Subsequent surface recombination losses can also be significantly reduced using a good passivation layer. The open-circuit voltage is determined by taking the limit of zero current in the general diode equation. This can be expressed as,


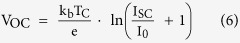


where I_0_ is the saturation current, K_b_ is the Boltzmann constant, and e is the electron charge. The prefactor (k_b_T_c_/e) represents the thermally induced potential. Finally, the fill factor is defined as the maximum electrical power output from the PV cell normalized by the product of the short-circuit current and open-circuit voltage,


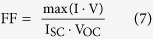


To determine the saturation current, an analytical expression can be obtained by considering the diffusion of carriers in the PN junction under zero external bias. For a finite size PN junction, this expression is as follows^81^,


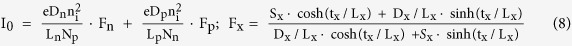


where is n_i_ the intrinsic carrier concentration, N_n_ is the n-type dopant concentration, N_p_ is the p-type dopant concentration, D_x_ is the carrier diffusivity, S_x_ is the surface recombination velocity, t_x_ is the thickness of the quasi-neutral regions, and L_x_ is the diffusion length. The subscripts n and p denote electrons and holes, respectively. The diffusion length can be defined in terms of a total recombination lifetime, 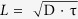. The recombination lifetime is an inverse summation over all recombination mechanisms including radiative recombination, Shockley-Read-Hall recombination, and Auger recombination^81^. The thickness of the p-type and n-type quasi-neutral regions are assumed equal and are obtained by taking the difference between the total thickness of the PV cell and the thickness of the space charge region which is expressed as^83^,


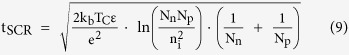


where *ε* is the permittivity of the PV cell taken in the long wavelength limit. Since the PV cell thickness is chosen independently in the radiative heat transfer model, if the thickness of the PV cell is smaller than the space charge region, the thicknesses of the p-type and n-type quasi-neutral regions are assumed to be negligible so that the PV cell consists entirely of a fully depleted region. In this limit, surface recombination becomes the dominant loss mechanism.

The electrical properties of the GaSb cell were obtained from literature^84^. It is assumed the GaSb cell is at a uniform temperature of 300K. The intrinsic carrier concentration, n_i_, is assumed to be 4.3 · 10^12^ cm^−3^. The electron and hole carrier concentrations are equal to N_n_ = N_p_ = 10^17^ cm^−3^. The recombination lifetimes are τ_R_ = 40 ns, τ_SHR_ = 10 ns, and τ_Au_ = 20 μs for radiative recombination, Shockley-Read-Hall recombination, and Auger recombination, respectively. The carrier diffusivities are D_n_ = 129 cm^2^/s and D_p_ = 39 cm^2^/s for electrons and holes, respectively. The surface recombination velocity is chosen to be S_n_ = S_p_ = 100 cm/s in accordance to previous studies^53^.

## Additional Information

**How to cite this article**: Tong, J. K. *et al.* Thin-film 'Thermal Well' Emitters and Absorbers for High-Efficiency Thermophotovoltaics. *Sci. Rep.* **5**, 10661; doi: 10.1038/srep10661 (2015).

## Supplementary Material

Supplementary Information
