# Supplementary material for: Thin-film ‘Thermal Well’ Emitters and Absorbers for High-Efficiency Thermophotovoltaics
Source: Sci Rep. 2015 Jun 1;5:10661. doi: 10.1038/srep10661 (PMC4649904; doi:10.1038/srep10661)
Supplement: Supplementary Information [file srep10661-s1.pdf]

Supplementary Information

Thin-film 'Thermal Well' Emitters and Absorbers for High-Efficiency Thermophotovoltaics

Jonathan K. Tong[1], Wei-Chun Hsu[1], Yi Huang[1], Svetlana V. Boriskina[1], and Gang Chen[1,*]

[1]*Department of Mechanical Engineering, Massachusetts Institute of Technology, Cambridge, MA 02139*

*Correspondence and requests for materials should be addressed to either S.V.B. or G.C. (emails: sborisk@mit.edu, gchen2@mit.edu)

Near-field formalism for thin-film structures. The thermal radiation model used in this study is a rigorous analytical formulation based on Rytov theory.[1–3] In this formulation, thermal emission is modelled electromagnetically as a volume of current sources. The spectral power density of these current sources is determined using the fluctuation-dissipation theorem and is defined as,

$$\left\langle J_i\left(r',\omega'\right)J_j^*\left(r'',\omega''\right)\right\rangle = \frac{\omega\varepsilon_0\varepsilon_r''\left(\omega\right)}{\pi}\theta\left(\omega,T\right)\cdot\delta\left(\omega'-\omega''\right)\delta\left(r'-r''\right)\delta_{ij} \quad \text{(S1)}$$

where ε_r'' is the imaginary component of permittivity, ε_0 is the vacuum permittivity, $\theta\left(\omega,T\right)$ is the mean energy of a Planck oscillator which generally includes the Bose-Einstein distribution and zero-point energy, r'/r" are source positions in the emitting medium, $\delta\left(r'-r''\right)$ implies locality of the materials response, and δ_{ij} implies isotropy of the material. In this study, the system is assumed to consist of a 1D multilayer stack of thin-films. The derivation thus follows from previous studies.[3,4] The heat flux from one medium to another is determined by calculating the Poynting vector in the film of interest using the Dyadic Green's functions to connect the current sources to a position within the film. To simplify the derivation, a cylindrical coordinate system is assumed. The heat flux from medium *m* to medium *n* takes the following form,

$$\begin{aligned} q_{mn}\left(z\right)=&\frac{k_v^2\cdot\theta\left(\omega,T_m\right)}{\pi^2}\cdot\mathrm{Re}\left\{i\varepsilon''\int dz'\int_0^\infty k_r dk_r\cdot\left[G_{E,mn,rr}\left(z,z',\omega\right)G^*_{H,mn,\theta r}\left(z,z',\omega\right)+\ldots\right.\right.\\ &\left.\left.\ldots+G_{E,mn,rz}\left(z,z',\omega\right)G^*_{H,mn,\theta z}\left(z,z',\omega\right)-G_{E,mn,\theta\theta}\left(z,z',\omega\right)G^*_{H,mn,r\theta}\left(z,z',\omega\right)\right]\right\} \end{aligned} \quad \text{(S2)}$$

where k_v is the vacuum wave vector, k_r is the in-plane wavevector, G_E is the Dyadic Green's function for the electric field, G_H is the Dyadic Green's function for the magnetic field, z is a position in film *n*, and z' is the source position in film *m*.

In this system, four types of waves can be defined based on the direction of propagation in film *n* (+/- z) and the direction of propagation in the emitting film *m* (+/- z'). These four types of waves are incorporated into the expression of the Dyadic Green's functions. From (S2), the expressions for the product of Dyadic Green's functions for the electric and magnetic fields can be separated according to the polarization relative to the plane of the film. For TM polarization, the expression is as follows,

$$
\begin{aligned}
&G_{E,mn,rr}G^{*}_{H,mn,\theta r}\left(k_r,z=z_n,\omega\right)+G_{E,mn,rz}G^{*}_{H,mn,\theta z}\left(k_r,z=z_n,\omega\right)=\ldots\\
&\quad\ldots=\frac{ik_{z,n}k_n^{*}}{8\mathrm{Re}\left(k_{z,m}\right)\mathrm{Im}\left(k_{z,m}\right)k_n\left|k_m\right|^2\left|k_{z,m}\right|^2}\cdot\ldots\\
&\quad\ldots\cdot\Bigg[\mathrm{Re}\left(k_{z,m}\right)\left(e^{2\mathrm{Im}\left(k_{z,m}\right)t_m}-1\right)\left(\left|k_{z,m}\right|^2+k_r^2\right)\cdot\left(-\left|A_n^{TM}\right|^2-A_n^{TM}B_n^{TM*}+A_n^{TM*}B_n^{TM}+\left|B_n^{TM}\right|^2\right)\cdot\ldots\\
&\quad\ldots\cdot i\cdot\mathrm{Im}\left(k_{z,m}\right)\left(e^{-i\cdot 2\mathrm{Re}\left(k_{z,m}\right)t_m}-1\right)\left(\left|k_{z,m}\right|^2-k_r^2\right)\cdot\left(A_n^{TM}C_n^{TM*}+A_n^{TM}D_n^{TM*}-B_n^{TM}C_n^{TM*}-B_n^{TM}D_n^{TM*}\right)\cdot\ldots\\
&\quad\ldots\cdot i\cdot\mathrm{Im}\left(k_{z,m}\right)\left(1-e^{i\cdot 2\mathrm{Re}\left(k_{z,m}\right)t_m}\right)\left(\left|k_{z,m}\right|^2-k_r^2\right)\cdot\left(A_n^{TM*}C_n^{TM}+B_n^{TM*}C_n^{TM}-A_n^{TM*}D_n^{TM}-B_n^{TM*}D_n^{TM}\right)\cdot\ldots\\
&\quad\ldots\cdot\mathrm{Re}\left(k_{z,m}\right)\left(1-e^{-2\mathrm{Im}\left(k_{z,m}\right)t_m}\right)\left(\left|k_{z,m}\right|^2+k_r^2\right)\cdot\left(-\left|C_n^{TM}\right|^2-C_n^{TM}D_n^{TM*}+C_n^{TM*}D_n^{TM}+\left|D_n^{TM}\right|^2\right)\Bigg]
\end{aligned}
\tag{S3}
$$

where t_m is the thickness of film m and the coefficients A, B, C, and D represent the amplitude of the four types of waves in the system. Similarly, for TE polarization,

$$
\begin{aligned}
&G_{E,mn,\theta\theta}G^{*}_{H,mn,r\theta}\left(k_r,z=z_n,\omega\right)=\ldots\\
&\quad\ldots=\frac{ik_{z,n}^{*}}{8\mathrm{Re}\left(k_{z,m}\right)\mathrm{Im}\left(k_{z,m}\right)\left|k_{z,m}\right|^2}\cdot\ldots\\
&\quad\ldots\cdot\Bigg[\mathrm{Re}\left(k_{z,m}\right)\left(e^{2\mathrm{Im}\left(k_{z,m}\right)t_m}-1\right)\cdot\left(\left|A_n^{TE}\right|^2-A_n^{TE}B_n^{TE*}+A_n^{TE*}B_n^{TE}-\left|B_n^{TE}\right|^2\right)\cdot\ldots\\
&\quad\ldots\cdot i\cdot\mathrm{Im}\left(k_{z,m}\right)\left(e^{-i\cdot 2\mathrm{Re}\left(k_{z,m}\right)t_m}-1\right)\cdot\left(A_n^{TE}C_n^{TE*}-A_n^{TE}D_n^{TE*}+B_n^{TE}C_n^{TE*}-B_n^{TE}D_n^{TE*}\right)\cdot\ldots\\
&\quad\ldots\cdot i\cdot\mathrm{Im}\left(k_{z,m}\right)\left(1-e^{i\cdot 2\mathrm{Re}\left(k_{z,m}\right)t_m}\right)\cdot\left(A_n^{TE*}C_n^{TE}-B_n^{TE*}C_n^{TE}+A_n^{TE*}D_n^{TE}-B_n^{TE*}D_n^{TE}\right)\cdot\ldots\\
&\quad\ldots\cdot\mathrm{Re}\left(k_{z,m}\right)\left(1-e^{-2\mathrm{Im}\left(k_{z,m}\right)t_m}\right)\cdot\left(\left|C_n^{TE}\right|^2-C_n^{TE}D_n^{TE*}+C_n^{TE*}D_n^{TE}-\left|D_n^{TE}\right|^2\right)\Bigg]
\end{aligned}
\tag{S4}
$$

A combination of the transfer matrix method and the scattering matrix method is used to recursively obtain analytical solutions for the coefficients A, B, C, and D. As an example, based on Fig. 1a of the main text, if the heat flux from medium 1 to medium 3 is desired, the coefficients take on the following form,

$$
\begin{aligned}
&A_3^{TM,TE}=\frac{e^{ik_{z,1}t_H}e^{ik_{z,2}g}t_{21}^{TM,TE}t_{23}^{TM,TE}}{\left(1+r_{21}^{TM,TE}r_{10}^{TM,TE}e^{2ik_{z,1}t_H}\right)\left(1+r_{23}^{TM,TE}r_{34}^{TM,TE}e^{2ik_{z,3}t_C}\right)\left(1-r_{20}^{TM,TE}r_{24}^{TM,TE}e^{2ik_{z,2}g}\right)}\cdot\frac{k_{z,1}}{k_{z,2}}\\
&B_3^{TM,TE}=r_{34}^{TM,TE}e^{2ik_{z,3}t_C}\cdot A_3^{TM,TE}\\
&C_3^{TM,TE}=r_{34}^{TM,TE}\cdot A_3^{TM,TE}\\
&D_3^{TM,TE}=\left(r_{34}^{TM,TE}\right)^2e^{2ik_{z,3}t_C}\cdot A_3^{TM,TE}
\end{aligned}
\tag{S5}
$$

where r and t are the Fresnel reflection and transmission coefficients for either an interface or a slab. For TM and TE polarizations, respectively, these coefficients are,

$$r_{mn}^{TM}=\frac{\varepsilon_n k_{z,m}-\varepsilon_m k_{z,n}}{\varepsilon_n k_{z,m}+\varepsilon_m k_{z,n}}$$
$$t_{mn}^{TM}=\frac{2k_{z,m}\sqrt{\varepsilon_m}\sqrt{\varepsilon_n}}{\varepsilon_n k_{z,m}+\varepsilon_m k_{z,n}} \quad \text{(S6)}$$

$$r_{mn}^{TE}=\frac{k_{z,m}-k_{z,n}}{k_{z,m}+k_{z,n}}$$
$$t_{mn}^{TE}=\frac{2k_{z,m}}{k_{z,m}+k_{z,n}} \quad \text{(S7)}$$

For a thin slab, the analytical expression for the reflection and transmission coefficients are,

$$r_{mo}^{TM,TE}=\frac{r_{mn}^{TM,TE}+r_{no}^{TM,TE}e^{2ik_{z,n}t_n}}{1+r_{mn}^{TM,TE}r_{no}^{TM,TE}e^{2ik_{z,n}t_n}}$$
$$t_{mo}^{TM,TE}=\frac{t_{mn}^{TM,TE}t_{no}^{TM,TE}e^{ik_{z,n}t_n}}{1+r_{mn}^{TM,TE}r_{no}^{TM,TE}e^{2ik_{z,n}t_n}} \quad \text{(S8)}$$

It should be noted that by using (S5), the calculated heat flux from medium 1 entering mediums 3 and 4 will be obtained. In order to isolate the contribution absorbed by medium 3, the portion of energy exiting medium 3 and entering medium 4 must be subtracted. To determine this heat flux, the same procedure is applied to obtain the Poynting vector from medium 1 to medium 4. In this case, medium 4 is assumed to be semi-infinite which thus simplifies the coefficients as shown,

$$A_4^{TM,TE}=\frac{e^{ik_{z,1}t_H}e^{ik_{z,2}g}e^{ik_{z,3}t_C}t_{21}^{TM,TE}t_{23}^{TM,TE}t_{34}^{TM,TE}}{\left(1+r_{21}^{TM,TE}r_{10}^{TM,TE}e^{2ik_{z,1}t_H}\right)\left(1+r_{23}^{TM,TE}r_{34}^{TM,TE}e^{2ik_{z,3}t_C}\right)\left(1-r_{20}^{TM,TE}r_{24}^{TM,TE}e^{2ik_{z,2}g}\right)}\cdot\frac{k_{z,1}}{k_{z,2}}$$
$$B_4^{TM,TE}=0 \quad \text{(S9)}$$
$$C_4^{TM,TE}=r_{34}^{TM,TE}\cdot A_4^{TM,TE}$$
$$D_4^{TM,TE}=0$$

To determine the net radiative heat transfer between mediums 1 and 3, the heat flux from medium 3 to medium 1 must also be found using the same procedure. By using the more general form of radiative transfer based on Rytov theory, no assumptions are made on the symmetry of the system. The model allows calculations using different emitter and PV cell materials, different film thicknesses, and different back side reflectors on the hot and cold sides.

Supplementary Figures:

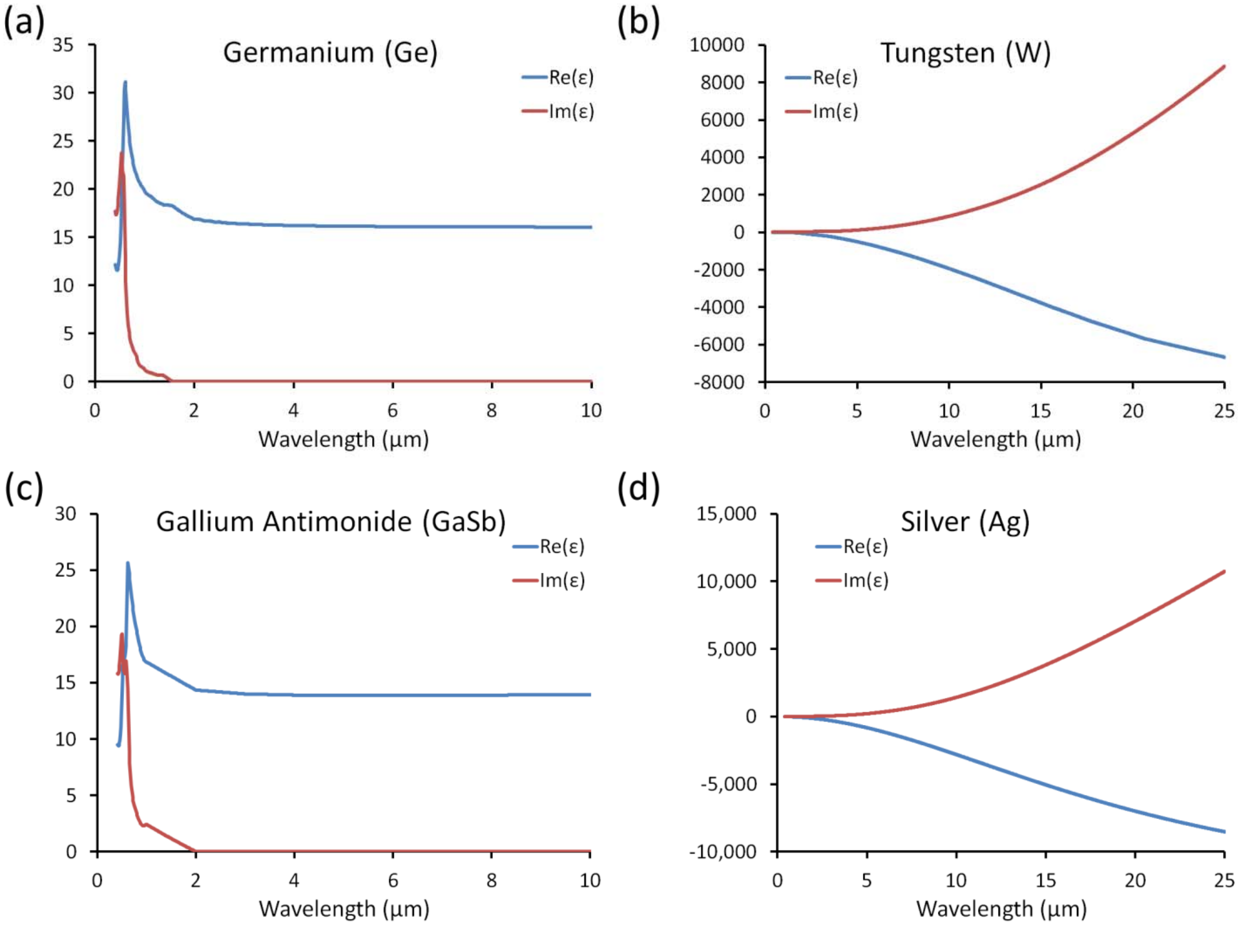

Figure S1: Optical constants of: (a) germanium (Ge), (b) tungsten (W), (c) gallium antimonide (GaSb), and (d) silver (Ag) obtained from the literature.[5] The optical constants for the perfect metal were taken in the long wavelength limit of silver and were assumed to be dispersionless for the wavelength range computed.

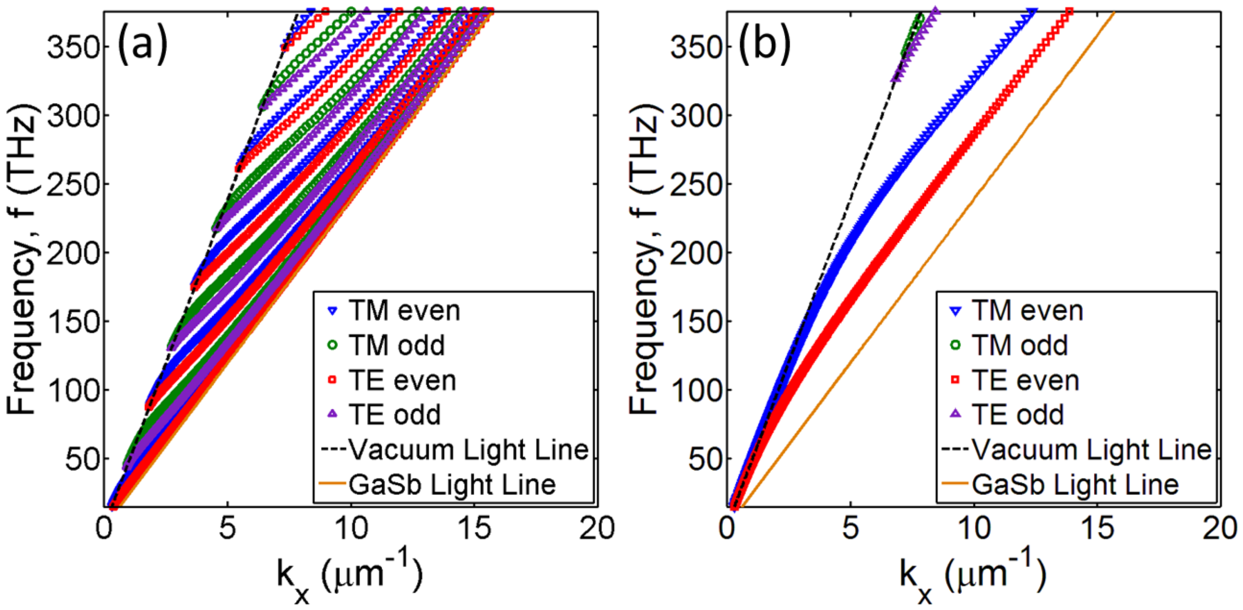

Figure S2: The dispersion for an isolated thin-film GaSb waveguide suspended in vacuum. (a) The dispersion for a 1 μm thick GaSb thin-film. (b) The dispersion for a 136 nm thick GaSb thin-film. As shown, variations in thickness can dramatically change the number of modes while simultaneously blue shifting the cut-off frequency of each waveguide mode. The first fundamental modes shown in (b) do not exhibit a cut-off frequency due to the symmetric nature of the waveguide. However, in the thin-film TPV system, the asymmetric nature of the waveguide system ensures a cut-off does exist.

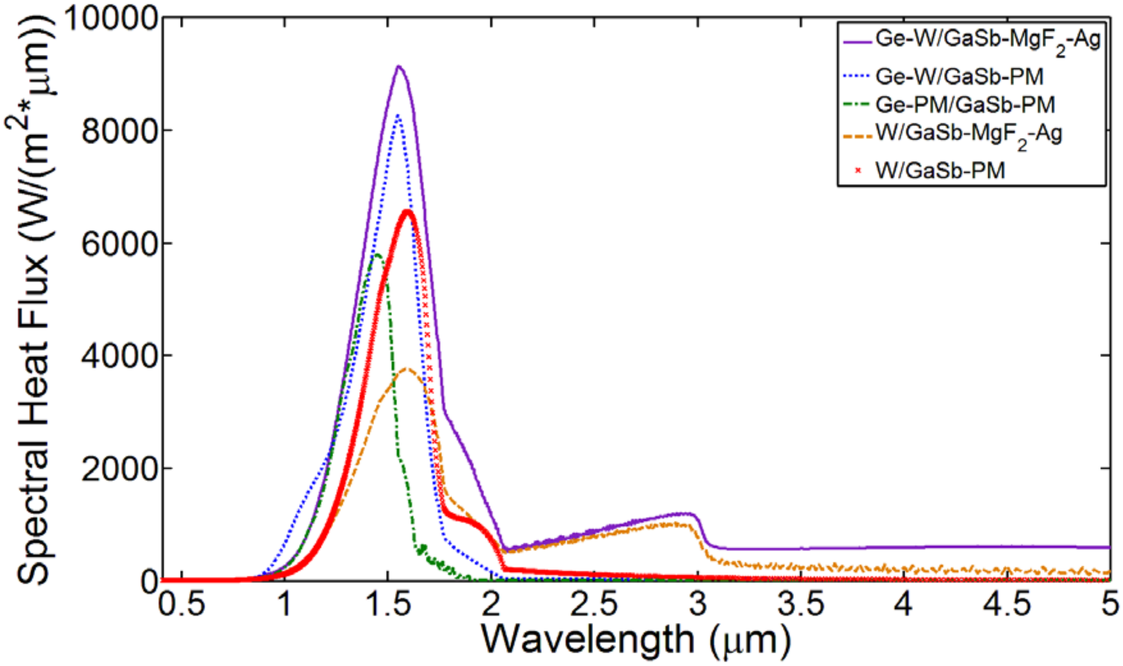

Figure S3: The spectral heat flux for the simulated cases presented in the main text. All cases are optimized to maximize the energy conversion efficiency. The thicknesses for all cases are: (1) Ge-W/GaSb-MgF_2-Ag: t_H = 119 nm, t_C = 100 nm, t_S = 1.25 μm, (2) Ge-W/GaSb-PM: t_H = 58 nm, t_C = 94 nm, (3) Ge-PM/GaSb-PM: t_H = 860 nm, t_C = 136 nm, (4) W/GaSb-MgF_2-Ag: t_C = 59nm, t_S = 750 nm, (5) W/GaSb-PM: t_C = 134 nm.

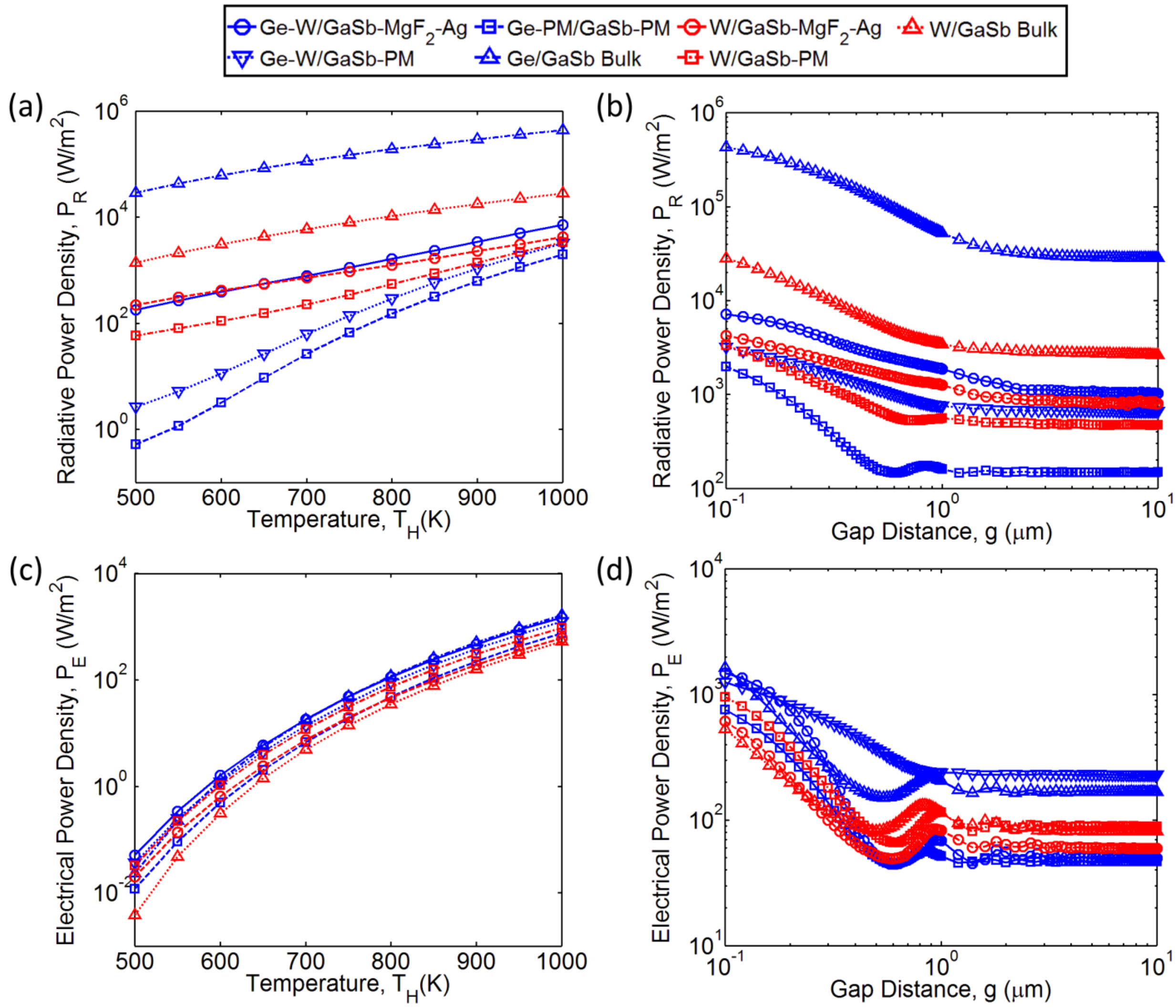

Figure S4: The radiative power density, P_R, which is defined as the net radiative transfer to the PV cell and underlying substrate for various combinations of the emitter material and back reflector material as a function of (a) the emitter temperature T_H and (b) the gap separation g between the emitter and the PV cell. The electrical power density, P_E, for various combinations of the emitter material and back reflector material as a function of (c) the emitter temperature T_H and (d) the gap separation g between the emitter and the PV cell.

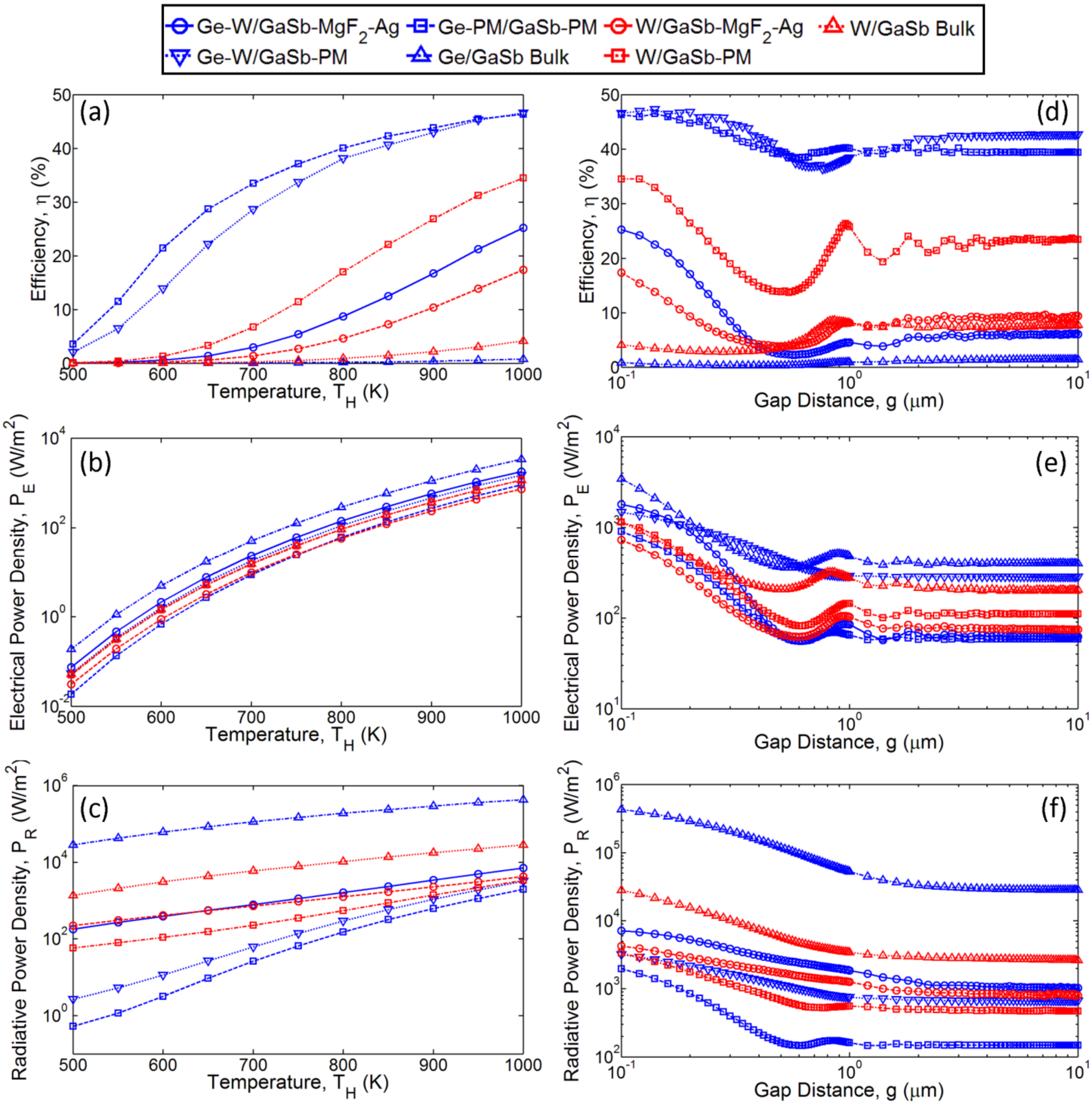

Figure S5: The predicted efficiency, η, the electrical power density, P_E, and the radiative power density, P_R, using the Shockley-Queisser formulation. All cases correspond to the main text using the same dimensions and parameters. Assuming a gap separation of g = 100 nm, the (a) efficiency, (b) electrical power density, and (c) radiative power density is plotted as a function of emitter temperature, T_H. Likewise, assuming an emitter temperature of T_H = 1000 K, the (d) efficiency, (e) electrical power density, and (f) radiative power density is plotted as a function of gap separation, g.

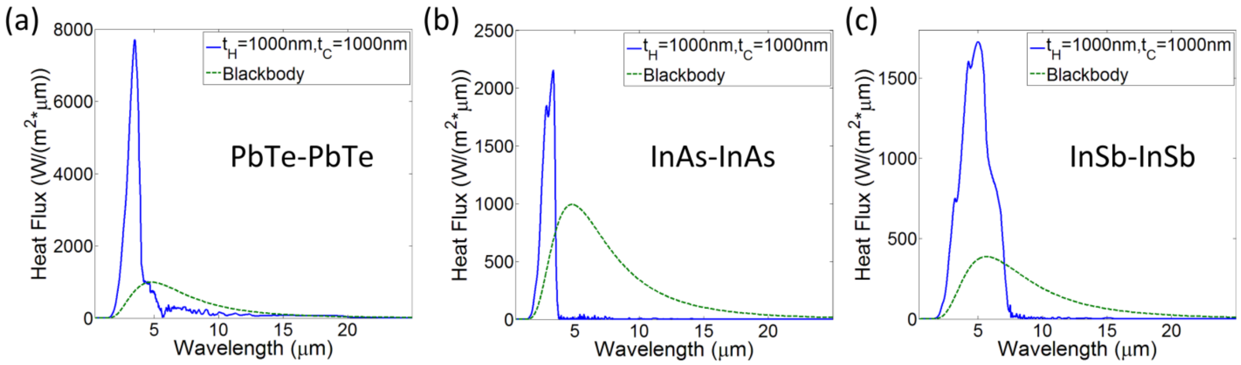

Figure S6: To show the generality of the 'thermal well' effect in manipulating thermal radiative transfer, several alternative material combinations were calculated, including: (a) lead telluride (PbTe) emitter and absorber assuming an emitter temperature of T_H = 600 K and absorber temperature of T_C = 300 K at a separation distance of 100 nm, (b) indium arsenide (InAs) emitter and absorber assuming an emitter temperature of T_H = 600 K and absorber temperature of T_C = 300 K at a separation distance of 100 nm, and (c) indium antimonide (InSb) emitter and absorber assuming an emitter temperature of T_H = 500 K and absorber temperature of T_C = 300 K at a separation distance of 100 nm. In all cases, the back reflector is chosen to be a perfect metal. It should be noted that these materials were not intended for use in high-temperature TPV applications, but rather to assess the potential spectral selectivity of thermal emission that could be achieved using the thermal well concept with different materials.

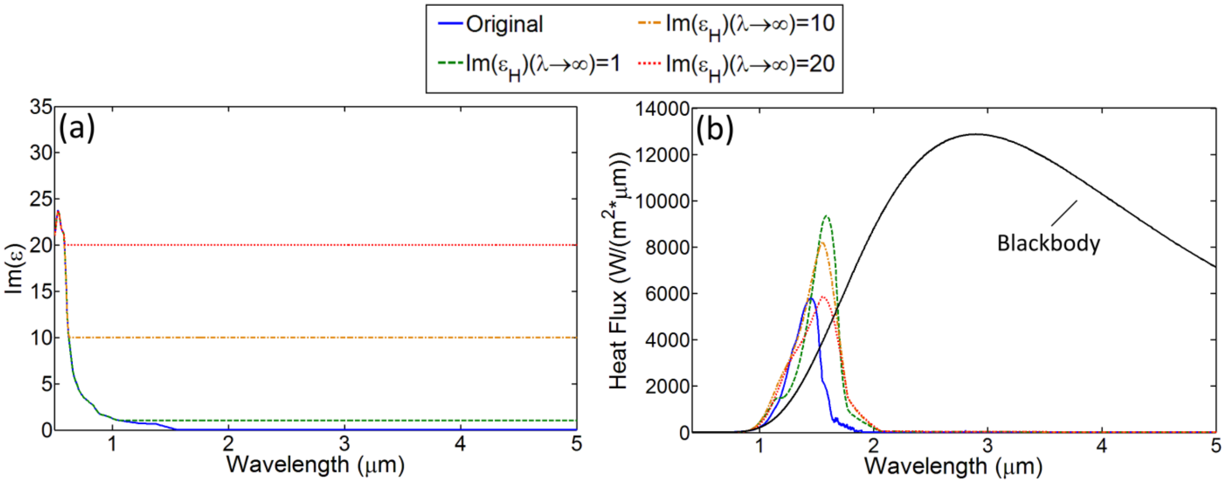

Figure S7: At high temperatures, the optical properties of Ge will change due to a combination of thermal expansion which decreases the electronic band-gap and a significant increase in the population of thermally excited free-carriers. These factors will both lead to emission at longer wavelengths. (a) To assess whether this will impact the spectral selectivity, the imaginary component of permittivity of germanium was artificially increased to simulate this effect. Each case was re-optimized to maximize efficiency. For $Im(\varepsilon_H \rightarrow \infty) = 1$: $t_H = 100$ nm, $t_C = 100$nm, $Im(\varepsilon_H \rightarrow \infty) = 10$: $t_H = 90$ nm, $t_C = 100$nm, $Im(\varepsilon_H \rightarrow \infty) = 20$: $t_H = 80$ nm, $t_C = 100$nm (b) The spectral radiative heat flux for a Ge thin-film emitter and a GaSb PV cell supported by a perfect metal still exhibits spectrally selective radiative transfer even for high material losses in the emitter. This can be explained by the inability of the GaSb thin-film to absorb long wavelength thermal radiation emitted by Ge due to the cutoff frequency of the lowest frequency mode. Although the spectral heat flux broadens and redshifts for higher material losses, the efficiency computed for each case is 39.8% for $Im(\varepsilon_H \rightarrow \infty) = 1$, 37.7% for $Im(\varepsilon_H \rightarrow \infty) = 10$, and 36% for $Im(\varepsilon_H \rightarrow \infty) = 20$.

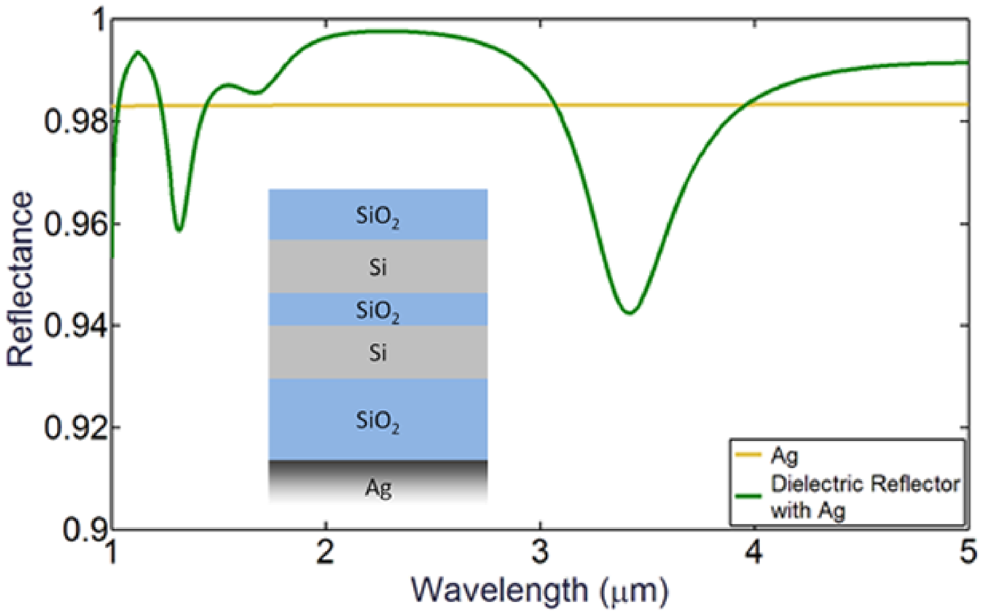

Figure S8: A preliminary design for a multilayer dielectric mirror was developed to show that it is possible to approach a near unity reflectance using real materials. This particular design is composed of silicon dioxide (SiO_2) and silicon (Si) supported by a silver (Ag) substrate with layer thicknesses from the top to the bottom of: 112 nm, 242 nm, 86 nm, 209 nm, and 308 nm. The dielectric mirror was specifically designed to improve the reflectance in the 2 to 3 μm wavelength range where parasitic absorption is more prevalent as shown in Figure S3. Compared to a Ag mirror, which exhibits a reflectance of about 98.3%, the dielectric mirror can achieve a reflectance as high as 99.7%. This may not appear to be a significant improvement, but, as discussed in the main text, a perfect metal back reflector and a Ag back reflector can lead to significant differences in the energy conversion efficiency. These results thus show that a simple multilayer dielectric stack can readily approach the perfect metal limit, suggesting it is possible to achieve high TPV efficiencies for a real system. It should be noted that this calculation was performed at normal incidence.

References

1. Rytov, S. M., Kravtsov, Y. A. & Tatarskii, V. I. *Principles of Statistical Radiophysics 3: Elements of Random Fields*. (Springer-Verlag Berlin Heidelberg, 1989).
2. Shen, S., Narayanaswamy, A. & Chen, G. Surface phonon polaritons mediated energy transfer between nanoscale gaps. *Nano Lett.* **9,** 2909–2913 (2009).
3. Francoeur, M., Mengüç, M. P. & Vaillon, R. Spectral tuning of near-field radiative heat flux between two thin silicon carbide films. *J. Phys. D. Appl. Phys.* **43,** 075501 (2010).
4. Francoeur, M., Pinar Mengüç, M. & Vaillon, R. Solution of near-field thermal radiation in one-dimensional layered media using dyadic Green's functions and the scattering matrix method. *J. Quant. Spectrosc. Radiat. Transf.* **110,** 2002–2018 (2009).
5. Palik, E. D. *Handbook of optical constants of solids*. (Academic Press, 1997).
